# Bacteriophages of Thermophilic ‘*Bacillus* Group’ Bacteria—A Systematic Review, 2023 Update

**DOI:** 10.3390/ijms25063125

**Published:** 2024-03-08

**Authors:** Piotr M. Skowron, Beata Łubkowska, Ireneusz Sobolewski, Agnieszka Zylicz-Stachula, Monika Šimoliūnienė, Eugenijus Šimoliūnas

**Affiliations:** 1 Department of Molecular Biotechnology, Faculty of Chemistry, University of Gdansk, Wita Stwosza 63, 80-308 Gdansk, Poland; ireneusz.sobolewski@ug.edu.pl (I.S.); a.zylicz-stachula@ug.edu.pl (A.Z.-S.); 2 Faculty of Health and Life Sciences, Gdansk University of Physical Education and Sport, K. Gorskiego 1, 80-336 Gdansk, Poland; beata.lubkowska@awf.gda.pl; 3 Department of Life and Environmental Sciences, University of Cagliari, 09124 Cagliari, Italy; 4 Department of Molecular Microbiology and Biotechnology, Institute of Biochemistry, Life Sciences Center, Vilnius University, Sauletekio Av. 7, LT-10257 Vilnius, Lithuania; monika.simoliuniene@gmc.vu.lt (M.Š.); eugenijus.simoliunas@bchi.vu.lt (E.Š.); 5 Department of Microbiology and Biotechnology, Institute of Bioscience, Life Sciences Center, Vilnius University, Sauletekio Av. 7, LT-10257 Vilnius, Lithuania

**Keywords:** thermophage, bacteriophage, bacteriophage TP-84, *Geobacillus*, *Parageobacillus*, *Geobacillus stearothermophilus*, *Geobacillus thermodenitrificans*, thermostable enzyme, *Bacillus*

## Abstract

Bacteriophages associated with thermophiles are gaining increased attention due to their pivotal roles in various biogeochemical and ecological processes, as well as their applications in biotechnology and bionanotechnology. Although thermophages are not suitable for controlling bacterial infections in humans or animals, their individual components, such as enzymes and capsid proteins, can be employed in molecular biology and significantly contribute to the enhancement of human and animal health. Despite their significance, thermophages still remain underrepresented in the known prokaryotic virosphere, primarily due to limited in-depth investigations. However, due to their unique properties, thermophages are currently attracting increasing interest, as evidenced by several newly discovered phages belonging to this group. This review offers an updated compilation of thermophages characterized to date, focusing on species infecting the thermophilic bacilli. Moreover, it presents experimental findings, including novel proteomic data (39 proteins) concerning the model TP-84 bacteriophage, along with the first announcement of 6 recently discovered thermophages infecting *Geobacillus thermodenitrificans*: PK5.2, PK2.1, NIIg10.1, NIIg2.1, NIIg2.2, and NIIg2.3. This review serves as an update to our previous publication in 2021.

## 1. Introduction

Bacteriophages, or phages, are viruses that infect bacteria. Earth is estimated to harbor over 10^31^ bacteriophages, making them the most abundant organisms on the planet. These viruses can be discovered and isolated wherever their host bacteria are present, spanning various environments such as soil, oceans, lakes, the bodies of humans and animals, as well as hot springs and hydrothermal vents [1].

In recent years, there has been a growing interest in utilizing bacteriophages and bacteriophage-derived components. Enzymes originating from phages have been indispensable tools in research since the early days of molecular biology. The proteins found in the capsids of bacteriophages have been utilized in the development of peptide vaccines using bacteriophage-based virus-like particles [2]. Biomaterials based on modified bacteriophages, used as drug delivery vehicles, have the potential to overcome the constraints associated with traditional drug delivery systems [3]. Such biomaterials present numerous benefits as drug carriers, including their high specificity for targeting bacterial cells, minimal toxicity, and the capacity for engineering to express specific proteins or peptides, thereby improving targeting precision and drug delivery efficiency [3].

Currently, an expanding number of research teams are investigating bacteriophages that infect thermophilic bacteria. These phages serve as an exceptionally abundant reservoir of novel proteins with distinctive properties and potential applications [4]. The characteristics of proteins obtained from thermophages, including inherent stability and functionality at elevated temperatures, provide significant benefits compared to their mesophilic counterparts in both industrial and molecular biology applications [4].

It is worth noting that the majority of commercially utilized viral enzymes originate from a restricted pool of cultivated viruses. Considering the vast diversity and abundance of viruses disclosed by metagenomic analysis, the exploration of genomes from uncultivated viruses emerges as a comprehensive and unexplored reservoir of new genes [4].

The growing interest in phage-based applications, advances in sequencing technology, and the prominence of microbiome research have prompted a significant shift in phage taxonomy [5,6]. The proposed approach advocates for the family level to represent a genomic unit of diversity, aligning taxonomy with the actual genetic composition of phages, and postulates the elimination of the order *Caudovirales* and the families *Myoviridae*, *Podoviridae*, and *Siphoviridae*, replacing them with monophyletic, genome-based families to create a taxonomy that can withstand future developments. However, the use of morphotype terminology, such as myovirus, podovirus, and siphovirus, remains relevant and is recommended for use in publications and annotated sequence records. The definition of species within this context considers two phages as part of the same species if their genomes exhibit more than 95% identity at the nucleotide level across their entire genome length, tested reciprocally.

For tailed phages, exemplified by the majority of *Geobacillus* (*G*.)/*Parageobacillus* (*P*.) phages, this reorganization of taxonomy has led to the introduction of the class *Caudoviricetes*, forming the foundation for defining new orders according to evolutionary relationships [5].

This review is an update of our previous article [7], which centered on bacteriophages, infecting a diverse group of thermophilic bacilli, comprising moderate thermophiles and thermophiles, such as *Geobacillus* and *Parageobacillus*, among others.

Prior to 2001, *Geobacillus* and *Parageobacillus* were classified as thermophilic variants of *Bacillus* spp. based on the analysis of 16S rRNA gene sequences [8]. Initially, the genus *Bacillus* encompassed five distinct phylogenetic groups, with some *Geobacillus*/*Parageobacillus* species falling into group 5 [8]. However, subsequent reclassification occurred, considering physiological characteristics, 16S rRNA gene sequences, fatty acid composition analyses, G-C contents, and DNA–DNA homology studies. *Geobacillus*/*Parageobacillus* species were then collectively reclassified as *Geobacillus* gen. nov. [9]. In 2016, Aliyu and colleagues conducted research leading to the separation of *Parageobacillus* from *Geobacillus* [10]. Their phylogenomic analysis of sixty-three *Geobacillus* strains with available genome sequences identified two clades based on differences in nucleotide base composition: clade I (comprised *Geobacillus* species) and clade II (comprised *Parageobacillus* species). Several species initially classified under *Geobacillus*, such as *P. caldoxylosilyticus*, *P. thermoglucosidasius*, *P. thermantarcticus*, *P. toebii*, and *P. genomospecies* 1 (NUB3621), were moved to *Parageobacillus* [10]. Further support for this classification was presented in 2020 by Najar and colleagues, who proposed including two additional species, *G. galactosidasius* and *G. yumthangensis*, in the genus *Parageobacillus* [11].

The aim of this review is to offer an updated compilation of the isolated and characterized bacteriophages that target this important group of thermophilic bacteria.

## 2. Review Data Search Methods

We performed a comprehensive search in PubMed, Scopus, and Web of Science databases to retrieve studies on thermophilic bacteriophages infecting thermophilic *Geobacillus* and *Parageobacillus.* Additionally, we searched bacteriophage collections and databases: DSMZ https://www.dsmz.de/ (accessed on 31 December 2023), ATTC (https://www.atcc.org/ (accessed on 31 December 2023), The *Bacillus* phage database http://bacillus.phagesdb.org/ (accessed on 31 December 2023), Virus-Host DB https://www.genome.jp/virushostdb (accessed on 31 December 2023) and PhageScope https://phagescope.deepomics.org/database (accessed on 31 December 2023).

We conducted the search by combining search terms related to thermophilic bacteriophages (thermophages, thermophilic viruses, thermophilic bacteriophages, *Siphoviridae*, *Siphoviruses*), thermostable enzymes (thermostable endolysins, thermostable polymerases, thermostable phage proteins), and thermophilic *Bacillus* species (*Geobacillus*, thermophilic *Bacillus*, *Bacillus stearothermophilus*, *Parageobacillus*, thermophilic bacteria).

The search was limited to publications published before 31 December 2023. Most of them were in English (with one exception).

This review includes all studies that addressed bacteriophages infecting moderately thermophilic and extremely thermophilic ‘*Bacillus* Group’ bacteria meeting the following criteria: they were research articles, review articles, conference abstracts, book chapters, or theses.

## 3. Chronological Review—2023 Update 

This review provides an update to our earlier publication on bacteriophages targeting the thermophilic ‘*Bacillus* group’ bacteria [7]. The update comprises a total of 72 species (including 13 recently discovered), documented in varying detail in original publications (Table 1). These bacteriophages target a range of thermophilic bacilli, including those previously categorized as *Bacillus*, such as ‘*G. stearothermophilus*’, *Acyclobacillus acidocaldarius*, *Acidithiobacillus caldus*, *B. caldotenax*, *G. caldolyticus*, *G. kaustophilus*, *Parabacillus thermoglucosidasius*, *G. icigianus*, *G. thermodenitrificans*, *A. pseudoalcaliphilus*, *A. bogoriensis*, *A. pseudofirmus*, *G. thermocatenulatus*, *B. cohnii*, as well as undefined *Bacillus* and *Aeribacillus* species.

## 4. Results and Discussion (Figure 1)

### 4.1. TP-84 (Caudoviricetes, Saundersvirus Tp84, Siphovirus morphotype, Host G. stearothermophilus)—2023 Update

Bacteriophage TP-84, discovered in 1952 in greenhouse soil, exhibits notable specificity for its host, particularly thriving in *G. stearothermophilus* strain 10 [18]. Other hosts, including (i) two unclassified thermophilic *Bacillus* strains (T-27 and 194); (ii) *G. stearothermophilus* strain 4 and 2184; (iii) two *Parageobacillus genomosp.* strains (NUB3621 and NUB3621R); and (iv) *G. thermoleovorans* 10 strain (accessible from Bacillus Genetic Stock Center; BGSCID 9A21) were found to support TP-84 infection to a varying extent [18,46]. Interestingly, *G. thermoleovorans* strain 10 (also known as *G. stearothermophilus* strain 10 [47]) is essentially identical to *G. stearothermophilus* strain 10 used by Saunders and Campbell [18], as the TP-84 bacterial host. It has a 52.6% GC genome, which is very close to the TP-84 GC content of 54.4%. It suggests a long history of this host–thermophage relationship. In contrast, *P. genomosp.* strains (NUB3621 and NUB3621R), which poorly support TP-84 growth, exhibit a distant GC content of 44.3%. The TP-84 bacteriophage temperature growth range of 31–80 °C [48], with an optimal range of 55–60 °C, aligns with the hosts’ growth range.

**Figure 1:**
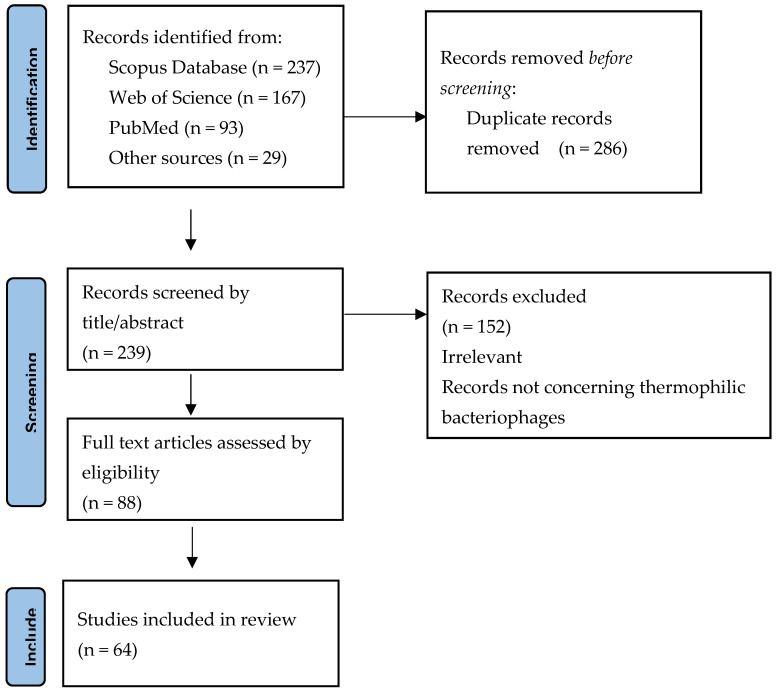
Systematic review flow diagram, detailing the database searches, the number of abstracts screened and the full texts retrieved.

The genome of TP-84 is a double-stranded DNA molecule consisting of 47,718 bp with a GC content of 43.9%. All 81 coding sequences (CDSs) are oriented in the same direction, and no RNA or DNA polymerase-coding genes were identified, indicating reliance on the host’s transcription and replication enzymes [49]. The genome, characterized by closely spaced and overlapping CDSs, contains putative promoter-like sequences and rho-independent terminators. In vitro translation experiments, cloning, and proteomic analysis have identified 73 (39 new, Table 2) TP-84-encoded proteins so far, including those involved in DNA replication/packaging, cell membrane disintegration, recombination processes, and envelope disintegration. Ten TP-84 genes have been cloned and expressed in *E. coli* so far, including endolysin (TP84_28) [50], depolymerase (glycosyl hydrolase (TP84_26) [48,51], major capsid protein (TP84_12), minor capsid protein (TP84_08), capsid portal protein (TP84_06), single-strand binding proteins (SSB) (TP84_54, TP84_63, TP84_66), helicase (TP84_59), and transcriptional regulator (TP84_52). The 39 new confirmed TP-84 proteins shown in Table 2 were determined by molecular cloning of the corresponding open reading frames (ORFs), transcription–translation in vitro, and LC-MS analysis. Further details are to be published elsewhere. Supplementary Figure S1 shows a genome map of TP-84 with updated proteomic confirmation of the bioinformatically characterized ORFs ([49] and this work). Only 8 ORFs, potentially coding for very small proteins, remain to be confirmed as active in protein biosynthesis.

### 4.2. AcaML1 (Unclassified Caudoviricetes, Myoviridae morphotype, Host Acidithiobacillus caldus ATCC 51756)

The existence of the temperate phage AcaML1 was discovered through the bioinformatic examination of the genome sequence of the moderately thermophilic *Acidithiobacillus caldus* ATCC 51756 [35]. The phage exhibits inducible characteristics and has the potential to infect and transfer genetic material to members of the *Acidithiobacillus* genus. Its genome consists of 59,353 bp with a GC content of 64.5%, surpassing the host’s average GC content of 61.6%. The genome contains 72 ORFs, organized into 10 clusters. Among these, 52.8% share sequence similarities with known proteins, 34.4% are hypothetical, and 12.8% bear no resemblance to any previously reported proteins. Gene clusters 1 and 2 encompass key proteins implicated in lysogeny establishment, as well as the regulation and control of the lysogeny–lytic switch, such as integrase, excisionase, regulators, primase, RNase endonuclease, and methyltransferases. Gene clusters 3 to 9 are dedicated to encoding products involved in viral particle formation and assembly, including procapsid shell and maturation protease, baseplate, contractile tail tube, tail fibers, DNA packaging components like terminase and portal protein, and factors contributing to host lysis for viral particle release, such as holin and endolysin. The terminal gene module in AcaML1 comprises three genes coding for an McrBC DNA restriction system, along with an associated modification enzyme (cytosine methylase), and two insertion sequences (IS5, IS21). The full genome sequence of AcaML1 is accessible in GenBank with the accession number JX507079.

### 4.3. GR1 (Unclassified Caudoviricetes, Siphovirus morphotype, Host G. stearothermophilus ATCC 10149)

Phage GR1 was isolated from roadside soil in Seoul using *G. stearothermophilus* ATCC 10149 as a host [41]. Transmission electron microscopy (TEM) revealed its distinctive features, including an icosahedral head with a diameter of 88 ± 9 nm (*n* = 10) and a noncontractile tail measuring 187 ± 44 nm (*n* = 10). Notably, GR1 exhibited strong infectivity against its host strain, forming large, clear plaques, and demonstrated specificity by only infecting three out of seven strains of *G. stearothermophilus*. GR1 displayed no infectivity towards other Gram-positive or Gram-negative bacteria, underscoring its high host specificity. The complete genome of GR1 consists of 79,387 base pairs of double-stranded DNA, with an average G + C content of 32.34%. It harbors 108 putative ORFs and one tRNA. Through BLASTN analysis, it was determined that GR1 is a novel phage with no genetically similar counterparts. This conclusion would corroborate with the very distance from its host GC content of 52.43%, which may indicate a relatively new establishment of this host–bacteriophage system and/or mosaic nature of the GR1, acquiring genes from various sources and adopting to infect *G. stearothermophilus*. The GR1 ORFs were classified into six distinct groups based on their functions: phage DNA packaging, phage structure, host lysis, nucleotide metabolism, additional functions, and hypothetical proteins. Noteworthy genes, such as integrase, imply that GR1 functions as a temperate phage. Additionally, genes encoding the major capsid protein, putative adhesin, terminase large subunit, and endolysin were identified in the phage. The endolysin coding gene was cloned and expressed in *E. coli*. Subsequently, the recombinant LysGR1 protein and its truncated derivatives were purified and subjected to biochemical and functional characterization. LysGR1 proved to be highly effective in eliminating *G. stearothermophilus* biofilms and exhibited notable thermostability, maintaining approximately 70% of its lytic activity, even after a 15 min incubation at 70 °C [41].

### 4.4. vB_GthS_PT9.1 (Unclassified Caudoviricetes, Siphovirus morphotype, Host Geobacillus thermodenitrificans)

**Figure 2:**
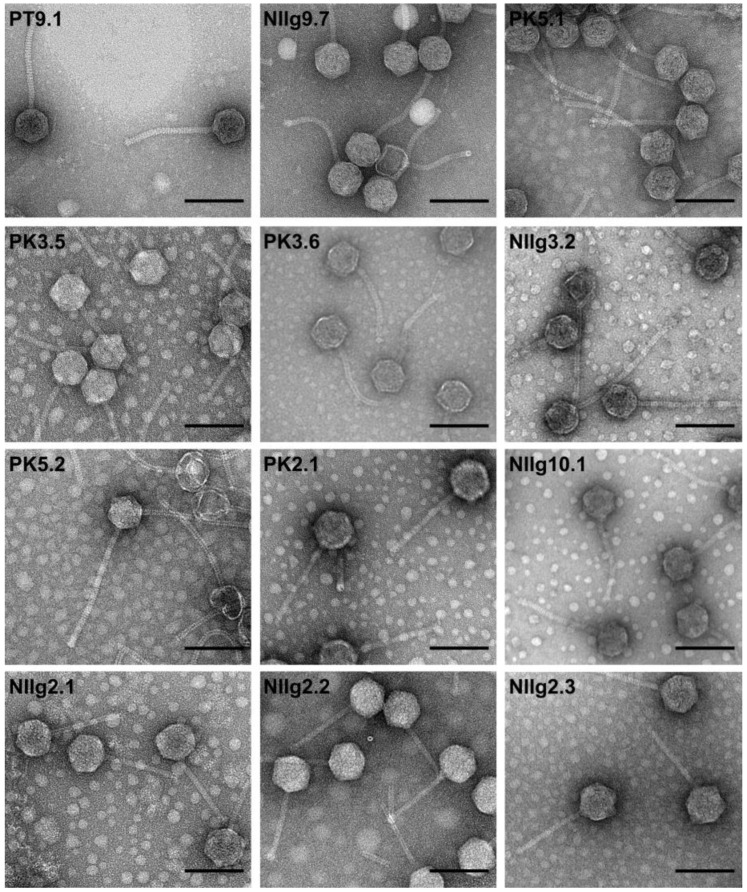
Transmission electron microscopy (TEM) analysis demonstrating the morphology of newly discovered thermophages infecting *G. thermodenitrificans*. Scale bar represents 100 nm.

Bacteriophage vB_GthS_PT9.1 (referred to here by its shorter name, PT9.1) was isolated from soil samples collected from compost heaps, using a soil-isolated *G. thermodenitrificans* strain PT-9 as a host [42]. The host range determination experiments demonstrated that out of 46 thermophilic *Bacillus*-group bacterial strains (including 41 local isolates), 9 strains of *G. thermodenitrificans* were sensitive to PT9.1. The phage infected its host cells from 45 to 80 °C. The PT9.1 formed plaques with a clear center (up to 3.2 mm in diameter) surrounded by an opaque halo zone (up to 9.4 mm in diameter), indicating the presence of phage-encoded enzyme, depolymerase. The TEM analysis revealed that the phage exhibited an icosahedral head with a diameter of 62.72 ± 2.34 nm, and a noncontractile tail of 143.15 ± 8.47 nm in length and 10.44 ± 1.03 nm in width, thus, apparently demonstrating the morphology of siphovirus (Figure 2). The genome of PT9.1 is a double-stranded (dsDNA) molecule consisting of 38,373 bp with a GC content of 43.9%. Similar to other dsDNA bacteriophages, the genome of PT9.1 is close-packed—94.2% of the genome is coded for proteins. The bioinformatics analysis revealed the presence of 75 probable protein-encoding genes and no genes for tRNA. Notably, all PT9.1 CDSs were oriented in the same direction. Bioinformatics analysis revealed that only ORF60 encoded unique proteins that had no reliable identity (E-values > 0.001) to the database entries. With the exception of gp03, which demonstrated 100% amino acid identity to hypothetical protein (WP_157778131.1) from *P. thermoglucosidasius*, all PT9.1 gene products showed the highest similarity to proteins from phages that infect bacteria from the genera *Geobacillus* (68), *Bacillus* (2), and *Paenibacillus* (1). Based on their homology to biologically defined proteins, 34 ORFs of PT9.1 were given a putative functional annotation. As observed in other siphoviruses, the PT9.1 genome appeared to have a modular organization, with genes for DNA packaging, structure/morphogenesis, lysis, phage–host interactions, and DNA replication/recombination/repair (DNA RRR) clustered together. It seems that temperate phages are more prone to modular evolution. Also, genes-coding proteins related to transcription, translation, nucleotide metabolism, and modification were presented in the genome of PT9.1. On the other hand, no RNA or DNA polymerase-coding genes were detected in the genome of this phage suggesting that PT9.1 most likely uses a number of DNA RRR proteins, including RNA or DNA polymerase, of the host cell [42]. Notably, none of the predicted gene products showed sequence homology with antibiotic resistance determinants or integration-related proteins. A proteomic analysis led to the experimental identification of three virion proteins, including major capsid protein (gp07), tape measure protein (gp16), and distal tail protein (gp17), which were predicted by bioinformatics approaches. A comparative total proteome comparison using the ViPTree web service demonstrated that PT9.1 was the most closely related to *Geobacillus* phage vB_GthS_NIIg9.7 (NIIg9.7). The nucleotide-based virus’s overall nucleotide sequence identity in between PT9.1 and NIIg9.7 was 82.5%, which suggests that these two phages are the members of a potential new viral genus [42]. A comparison of functional genome maps of *(Para)geobacillus* bacteriophages PT9.1, NII9.7, PK5.1, PK3.5, PK3.6, and NII3.2 is shown in Supplementary Figure S2.

### 4.5. vB_GthS_NIIg9.7 (Unclassified Caudoviricetes, Siphovirus morphotype, Host G. thermodenitrificans)

Bacteriophage vB_GthS_NIIg9.7 (referred to here by its shorter name, NIIg9.7) was isolated from soil samples collected from compost heaps, using a soil-isolated *G. thermodenitrificans* strain NIIg-9 as a host [42]. The host range determination experiments revealed that phage NIIg9.7 infected nine *G. thermodenitrificans* strains (out of 46 thermophilic Bacillus-group bacterial strains tested). The phage propagation temperature was from 50 to 78 °C. The plaques formed by NIIg9.7 had a clear center (up to 3.0 mm in diameter) surrounded by an opaque halo zone (up to 11.4 mm in diameter). Based on the morphological characteristics, NIIg9.7 is a siphovirus characterized by an isometric head (63.81 ± 3.95 nm in diameter) and a noncontractile tail (143.16 ± 4.19 nm in length, and 9.93 ± 1.62 nm in width) (Figure 2). The NIIg9.7 contains dsDNA consisting of 39,016 bp with a GC content of 44.4%. The 93.9% of the genome is coded for proteins. The 76 probable protein-encoding genes and no genes for tRNA were detected in the genome of NIIg9.7. With the exception of ORF40 encoding a ribbon–helix–helix domain-containing protein, all other NIIg9.7 ORFs have been predicted to be transcribed from the same DNA strand. Based on the results of bioinformatics analysis, only two NIIg9.7 ORFs, which are ORF41 and ORF67, encoded unique proteins that had no reliable identity to the database entries. With the exception of gp35 and gp45, which demonstrated 61% and 64% amino acid identity to hypothetical protein (WP_098417325.1) from *Bacillus cereus* and DUF559 domain-containing protein (WP_236934102.1) from *G. thermodenitrificans*, accordingly, all NIIg9.7 gene products showed similarity to viral proteins. The highest similarity was to proteins from phages that infect bacteria from the genera *Geobacillus* (66), *Bacillus* (3), *Brevibacillus* (1), *Virgibacillus* (1) and *Thermus* (1). Based on their homology to biologically defined proteins, 35 ORFs of NIIg9.7 were functionally annotated. The genes-coding proteins for DNA packaging, structure/morphogenesis, lysis, phage–host interactions, DNA replication/recombination/repair, transcription, translation, and nucleotide metabolism/modification were detected in the genome of NIIg9.7. On the other hand, no RNA or DNA polymerase-coding genes and no genes-coding antibiotic resistance determinants or integration-related proteins were detected in the genome of NIIg9.7. Mass spectrometry analysis led to the experimental identification of four virion proteins, including major capsid protein (gp06), tape measure protein (gp15), distal tail protein (gp16), and tail fiber protein (gp17) that were predicted by bioinformatics approaches. A comparative total proteome comparison using the ViPTree web service demonstrated that the closest relative of NIIg9.7 was *Geobacillus* phage PT9.1. The nucleotide-based virus overall nucleotide sequence identity of NIIg9.7 vs. PT9.1 was 82.5% [42]. A comparison of functional genome maps of *(Para)geobacillus* bacteriophages PT9.1, NII9.7, PK5.1, PK3.5, PK3.6, and NII3.2 is shown in Supplementary Figure S2.

### 4.6. vB_GthS_PK5.1 (Unclassified Caudoviricetes, Siphovirus morphotype, Host G. thermodenitrificans)

Bacteriophage vB_GthS_PK.1 (referred to here by its shorter name, PK5.1) was isolated from soil samples collected from compost heaps, using a soil-isolated *G. thermodenitrificans* strain PK-5 as a host [42]. Phage PK5.1 demonstrated a narrow host range: it infected only two *G. thermodenitrificans* strains (out of 46 thermophilic *Bacillus*-group bacterial strains tested). Phage propagation temperature was from 48 to 80 °C. The PK5.1 formed plaques with a clear center (up to 3.7 mm in diameter) surrounded by an opaque halo zone (up to 7.2 mm in diameter). The TEM analysis revealed siphovirus morphotype: PK5.1 possesses an icosahedral head with a diameter of 62.91 ± 3.20 nm, and a tail of 145.74 ± 14.88 nm in length and 10.10 ± 1.42 nm in width (Figure 2). The genome of PK5.1 consists of 38,161 bp dsDNA with a GC content of 43.6%. Also, 92.4% of the genome is coded for proteins. The bioinformatics analysis revealed the presence of 64 probable protein-encoding genes and no genes for tRNA. Notably, all CDSs were oriented in the same direction. All PK5.1 ORFs encoded proteins that had a reliable identity to the database entries. With the exception of gp33, which demonstrated 65% amino acid identity to hypothetical protein (WP_043904680.1) from *Parageobacillus genomosp*. 1, all PK5.1 gene products had reliable identity to viral proteins: it showed the highest similarity to proteins from phages that infect bacteria from the genera *Geobacillus* (59), *Bacillus* (3), and *Lactobacillus* (1). In total, 37 ORFs of PK5.1 were given a putative functional annotation. Genes coding for DNA packaging, structure/morphogenesis, lysis, phage–host interactions, DNA RRR, transcription, translation, nucleotide metabolism, and modification proteins were presented in the genome of PK5.1. On the other hand, no RNA or DNA polymerase-coding genes as well as no genes-coding antibiotic resistance determinants or integration-related proteins were detected. Proteomic analysis led to the identification of three virion structure/morphogenesis-related proteins including Clp protease (gp04), major capsid protein (gp05), tape measure protein (gp14), and structural protein (gp16). A comparative total proteome comparison using the ViPTree web service demonstrated that PK5.1. was in between Geobacillus phages PT9.1-NIIg9.7 and PK3.5-PK3.6. The nucleotide-based virus overall nucleotide sequence identity between PK5.1 and its closest relatives revealed a 57.9%, 56.8%, 54.4%, and 48.5% identity with PK3.5, PK3.6, NIIg9.7, and PT9.1, accordingly. These results suggest that *Geobacillus* phage PK5.1 has no close identity to phages deposited in the NCBI database to date and potentially represents a new genus within siphoviruses. A comparison of functional genome maps of *(Para)geobacillus* bacteriophages PT9.1, NII9.7, PK5.1, PK3.5, PK3.6, and NII3.2 is shown in Supplementary Figure S2.

### 4.7. vB_GthS_PK3.5 (Unclassified Caudoviricetes, Siphovirus morphotype, Host G. thermodenitrificans)

Phage vB_GthS_PK3.5 (PK3.5) was isolated from soil samples collected from compost heaps, using a *G. thermodenitrificans* strain PK-3 as a host [42]. PK3.5 infected 4 *G. thermodenitrificans* strains (out of 46 thermophilic *Bacillus*-group bacterial strains tested). The phage demonstrated an ability to infect its host cells from 50 to 78 °C. Clear plaques (up to 1.5 mm in diameter) were surrounded by a turbid halo zone (up to 4.92 mm in diameter). Based on the results of TEM analysis, PK3.5 is a siphovirus characterized by an icosahedral head with a diameter of 65.18 ± 3.27 nm, and a tail of 149.98 ± 15.01 nm in length and 10.84 ± 1.92 nm in width (Figure 2). PK3.5 contains a genome of 38,788 bp dsDNA with a GC content of 43.5%. The coding capacity of the genome is 92.7%. A total number of 76 probable protein-encoding genes and no genes for tRNA were detected in the genome of PK3.5. All ORFs have been predicted to be transcribed from the same DNA strand. Notably, the vast majority (71 out of 76) PK3.5 ORFs encoded proteins that had a reliable identity to the database entries. For PK3.5 ORFs that encoded proteins with matches to those in other sequenced viral genomes, it showed the highest similarity to proteins from phages that infect bacteria from the genera *Geobacillus* (63), *Lysinibacillus* (1), and *Psychrobacillus* (1), *Ochrobactrum* (1), and *Streptococcus* (1). In total, 37 ORFs of PK3.5 were given a putative functional annotation including genes coding for DNA packaging, structure/morphogenesis, lysis, phage–host interactions, DNA RRR, transcription, translation, nucleotide metabolism, and modification. On the other hand, no RNA or DNA polymerase-coding genes were detected. Also, no predicted genes-coding antibiotic resistance determinants or integration-related proteins were detected in the genome of PK3.5. Proteomic analysis of the structural proteins of phage PK3.5 virions using a modified filter-aided sample preparation (FASP) protocol, followed by Liquid Chromatography with Tandem Mass Spectrometry (LC-MS/MS), led to the identification of five virion structural proteins including major capsid protein (gp05), putative tail component (gp09), tape measure protein (gp14), distal tail protein (gp15) and putative tail fiber protein (gp16). Neighbor-joining tree analysis based on the alignment of the amino acid sequences of major capsid protein, terminase large subunit, tape measure protein, and helicase, as well as a comparative total proteome comparison using the ViPTree web service, demonstrated that PK3.5 was the most closely related to *Geobacillus* phage vB_GthS_PK3.6 (PK3.6). The nucleotide-based virus’s overall nucleotide sequence identity in between PK3.5 and PK3.6 was 48.5%. These results suggest that these two phages are the members of a potential new viral genus within siphoviruses [42]. A comparison of functional genome maps of *(Para)geobacillus* bacteriophages PT9.1, NII9.7, PK5.1, PK3.5, PK3.6, and NII3.2 is shown in Supplementary Figure S2.

### 4.8. vB_GthS_PK3.6 (Unclassified Caudoviricetes, Siphovirus morphotype, Host G. thermodenitrificans)

Bacteriophage vB_GthS_PK3.6 (referred to here by its shorter name, PK3.6) was isolated from soil samples collected from compost heaps, using a soil-isolated *G. thermodenitrificans* strain PK-3 as a host [42]. Phage infected only 2 out of 46 thermophilic *Bacillus*-group bacterial strains tested (including 41 local isolates). Phage propagated in a temperature range from 50 to 80 °C. The PK3.6 formed plaques with a clear center (up to 3.0 mm in diameter) surrounded by an opaque halo zone (up to 8.4 mm in diameter). The bacteriophage demonstrated the morphology of siphovirus: it has an icosahedral head with a diameter of 66.93 ± 4.24 nm, and a noncontractile tail of 137.32 ± 5.75 nm in length and 9.41 ± 1.84 nm in width (Figure 2). The PK3.6 contains dsDNA (38,405 bp) with a GC content of 44.8% and a coding capacity of 92.0%. In total, 68 probable protein-encoding genes and no genes for tRNA were detected in the genome of PK3.6. Notably, all CDSs were oriented in the same direction. All PK3.6 ORFs encoded proteins that had a reliable identity to the database entries. Notably, all CDSs were oriented in the same direction. All PK5.1 ORFs encoded proteins that had reliable identity to viral proteins deposited in NCBI database: it showed the highest similarity to proteins from phages that infect bacteria from the genera *Geobacillus* (59), *Anoxybacillus* (2), *Bacillus* (1), and *Psychrobacillus* (1), *Enterococcus* (1), *Lactococcus* (1), and *Sporosarcina* (1). In total, 36 ORFs of PK3.6 were given a putative functional annotation. Genes coding for DNA packaging, structure/morphogenesis, lysis, phage–host interactions, DNA RRR, transcription, translation, nucleotide metabolism, and modification proteins were detected in the genome of PK3.6. In contrast, none of the predicted gene products showed sequence homology with RNA or DNA polymerase-coding genes, as well as no genes-coding antibiotic resistance determinants or integration-related proteins, were presented. Three virion proteins, including major capsid protein (gp05), tape measure protein (gp14), and tail fiber protein (gp16), were experimentally evaluated by MS analysis. Neighbor-joining tree analysis based on the alignment of the amino acid sequences of the individual proteins most often used for the analysis of the evolutionary relationships among bacteriophages, as well as a comparative total proteome comparison using the ViPTree web service demonstrated that PK3.6 was the most closely related to *Geobacillus* phage PK3.6. The nucleotide-based virus’s overall nucleotide sequence identity in between PK3.6 and its closest relative, that is, PK3.5, was 84.2%. A comparison of functional genome maps of *(Para)geobacillus* bacteriophages PT9.1, NII9.7, PK5.1, PK3.5, PK3.6, and NII3.2 is shown in Supplementary Figure S2.

### 4.9. vB_PtoS_NIIg3.2 (Unclassified Caudoviricetes, Siphovirus morphotype, Host G. thermodenitrificans)

Bacteriophage vB_PtoS_NIIg3.2 (referred to here by its shorter name, NIIg3.2) was isolated from the compost heaps using the enrichment of phages in the source material technique, with the local isolate *Parageobacillus toebii* strain NIIg-3 as a host [43]. In addition, four *G. thermodenitrificans* strains (PT-4, NIIg-2, PK-11, and PK-3) were sensitive to NIIg3.2 (out of 46 thermophilic *Bacillus*-group bacterial strains tested). The phage infected its host cells from 50 to 80 °C. The NIIg3.2 formed plaques with a clear center (up to 0.6 mm in diameter) surrounded by an opaque halo zone (up to 2 mm in diameter). TEM analysis revealed siphovirus morphology: phage is characterized by an icosahedral head with a diameter of 62.08 ± 3.96 nm, and a long, noncontractile tail of 218.37 ± 12.53 nm in length, and 10.44 ± 1.03 nm in width (Figure 2). Phage NIIg3.2 has a dsDNA genome consisting of 38,970 bp with a GC content of 42.2% and a coding capacity of 94.6%. Phage contains 71 probable protein-encoding genes and no genes for tRNA. With the exception of ORF24-ORF26, encoding proteins potentially related to the lysogenic module of NIIg3.2, all other ORFs were oriented in the same DNA strand. Based on the results of bioinformatics analysis, only ORF31 encoded unique proteins that had no reliable identity to the NCBI database entries. Interestingly, even 14 ORFs encoded proteins with reliable identity exceptionally for bacterial proteins. The NIIg3.2 ORFs that encoded proteins with reliable identity to viral homologs showed the highest similarity to proteins from phages that infect bacteria from the broad-range genera including *Geobacillus* (32), *Bacillus* (11), *Thermus* (4), *Clostridium* (2), *Paenibacillus* (2), *Virgibacillus* (2), *Anoxybacillus* (1), *Brevibacillus* (1), *and Staphylococcus* (1). Based on homology to biologically defined proteins, 29 ORFs of NIIg3.2 were given a putative functional annotation including genes coding for DNA packaging, structure/morphogenesis, phage–host interactions, lysis, and DNA replication/repair. Also, the lysogeny module of NIIg3.2 was found downstream of the lysis cassette, whereas gp50 encoded the potential auxiliary metabolic gene (AGM) related to pathogenicity–YopX family protein containing YopX (pfam09643) conserved domain. No RNA or DNA polymerase-coding genes were detected in the genome of this phage. Proteomic analysis confirmed five NIIg3.2 structural proteins identified by comparative genomics and/or HMM profile comparisons: prohead protease (gp05), major capsid protein (gp06), tape measure protein (gp15), tail hube protein (gp16), and tail fiber protein (gp17). Neighbor-joining tree analysis based on the alignment of the amino acid sequences of major capsid protein, terminase large subunit, portal protein, and tape measure protein as well as a comparative total proteome comparison using the ViPTree web service revealed that NIIg3.2 had no close phylogenetic relationships to viral homologs to date. The nucleotide-based virus overall nucleotide sequence identity between NIIg3.2 and its closest relatives was quite low and ranged from 15.5% (NIIg3.2 vs. PK5.1) to 9.6% (NIIg3.2 vs. PK3.6) indicating that NIIg3.2 represents an evolutionarily distant lineage within the bacterial viruses. A comparison of functional genome maps of *(Para)geobacillus* bacteriophages PT9.1, NII9.7, PK5.1, PK3.5, PK3.6, and NII3.2 is shown in Supplementary Figure S2.

### 4.10. vB_GthS_PK5.2, vB_GthS_PK2.1, vB_GthS_NIIg10.1, vB_GthS_NIIg2.1, vB_GthS_NIIg2.2, vB_GthS_NIIg2.3 (Unclassified Caudoviricetes, Siphovirus morphotype, Host G. thermodenitrificans)

Bacteriophages vB_GthS_PK5.2, vB_GthS_PK2.1, vB_GthS_NIIg10.1, vB_GthS_NIIg2.1, vB_GthS_NIIg2.2, and vB_GthS_NIIg2.3 (referred to here by their shorter names, PK5.2, PK2.1, NIIg10.1, NIIg2.1, NIIg2.2, and NIIg2.3, accordingly) were isolated from soil samples collected from compost heaps at Vilnius University Botanical Garden, Vingis Park, Vilnius, Lithuania, using the enrichment of phages in the source material technique. *G. thermodenitrificans* strains PK-5, PK-2, and NIIg-10 were used as the host for PK5.2, PK2.1, and NIIg10.1 isolation, propagation, and phage growth experiments, accordingly. *G. thermodenitrificans* strain NIIg-2 was used as a host for NIIg2.1, NIIg2.2, and NIIg2.3 experiments. TEM analysis revealed that all phages are siphoviruses characterized by an isometric head and a noncontractile tail (Figure 2). All bacteriophages contain dsDNA. Complete genome sequences and annotation of genomic DNA of phages PK5.2 and PK2.1 have now been deposited in the NCBI database under accession numbers OP341629.1 and OP341625.1, accordingly. Preliminary bioinformatics and phylogenetics analysis suggest that phage PK2.1 is closely related to *Geobacillus* phage TP-84; meanwhile, PK5.2 has no close viral homologs to date and potentially represents a new genus within siphoviruses.

## 5. Perspective

Thermophilic bacteriophages, with their unique properties and adaptability to extreme temperatures, hold significant promise in biotechnology. Their diverse applications, from bioprocessing to antimicrobial strategies and bioremediation, underscore their potential as valuable tools in various biotechnological processes. Thus, from a practical perspective, thermophilic *Bacillus* species are key bacterial entities in industrial fermentation processes—along with their bacteriophages. In general, thermophages, not only those from the ‘*Bacillus* group’, present a novel reservoir of diversified genetic material and enzymes, holding significant potential for applications in both industry and scientific research [7]. For example, *G. stearothermophilus* is a spore-forming Gram-positive bacterium that causes flat sour spoilage in low-acid canned foods. To address this problem, *G. stearothermophilus*-infecting phage GR1 was isolated from the soil, and its endolysin LysGR1 was characterized [41]. Considering the high thermal stability, broad lytic activity, and biofilm reduction efficacy of LysGR1 and its enzymatically active domain, it is suggested that these enzymes could act as promising biocontrol agents against *G. stearothermophilus* and foodborne pathogens [41]. Thermophilic bacteriophages may be useful for bioremediation purposes, particularly in the removal of thermotolerant bacterial contaminants from environmental sources, biofilms, and industrial wastewater, just like their mesophilic equivalents [52,53] or, to the contrary, they may negatively affect high-temperature biological treatments of raw sewage sludge by infecting employed thermophilic bacteria [4,54]. Despite advances in modern technologies, various foodborne outbreaks have continuously threatened food safety. The overuse and misuse of antibiotics have escalated this threat due to the prevalence of multidrug-resistant (MDR) pathogens. Therefore, the development of new methodologies for controlling microbial contamination is extremely important to ensure food safety [55]. As an alternative to conventional antibiotics, bacteriophages and their derived endolysins have emerged as promising, effective, and safe antimicrobial agents [56]. Thermophages have also shown potential as antimicrobial agents against thermotolerant pathogens. For example, the thermophilic bacteriophage TSP4 has been investigated for its antibacterial activity against antibiotic-resistant strains of *Staphylococcus aureus* [57]. Another health-related application includes the potential for the construction of novel types of vaccine nanocarriers and drug delivery vehicles by employing SMV1 coat protein from unclassified *Bicaudaviridae* SMV1, infecting *Sulfolobus monocaudavirus*. The protein *has shown* in vivo stability without inflammatory response in mice and human intestinal organoids [58]. Thermophilic or thermotolerant bacteriophages may be used to control bacterial contaminants, including both high- and moderate-temperature food processing environments. For instance, the thermophilic bacteriophage DZ1 may be used as a biocontrol agent for *Bacillus cereus* contamination during industrial food production [59]. These novel biological entities and their enzymes are increasingly being explored for their potential to prevent and eradicate bacterial contaminants, even within the intricate settings of foods and food processing facilities [55,56,60]. Bacteriophage-coded thermostable enzymes are also important tools in molecular biology technologies and molecular diagnostics, such as, for example, (i) 3173 DNA polymerase cloned as a metagenomic fragment from ‘Pyrovirus’ (Yellowstone) with unique features of proofreading DNA-dependent DNA polymerase activity and reverse transcriptase activity with optimum at 77 °C and half-life of 11 min. at 94 °C. The enzyme is used as ‘one-enzyme’ RT-PCR [61]; (ii) Magma DNA polymerase–recombinant chimera of metagenomic thermophage DNA polymerase and thermophilic bacteria *Thermus aquaticus* Taq DNA polymerase, exhibiting an extremely low replication error level of 1 per 10^6^ nucleotides, and are thus used for high fidelity PCR [62]; (iii) NrS-1 primase-polymerase from unclassified siphovirus NrS-1–multifunctional enzyme possessing DNA polymerase, primase, and helicase activity with optimum at 50 °C. Used for primer-less DNA synthesis for whole genome amplification [63]; and (iv) TS2126 RNA/ssDNA ligase from unclassified *Thermus* bacteriophage Ph2119) exhibits very high specificity—30× higher than mesophilic T4 bacteriophage RNA ligase with optimum at 65 °C [64]. Commercialized for cDNA ligation and circularization of linear nucleic acids.

The increasing number of discoveries of thermophages, including those infecting thermophilic ‘*Bacillus* group’ species, provides a more complete understanding of thermophiles’ biology, the mechanisms of biochemical adaptations needed for life in high temperatures, and the evolution of thermophilic host–bacteriophage relationships. Especially interesting is the recent characterization of numerous thermophages that were isolated from compost heaps using *G. thermodenitrificans* and *Parageobacillus* strains as hosts for bacteriophage propagation [42,43].

## Figures and Tables

**Table 1:** Classification and major data describing ‘*Bacillus* group’ thermophilic bacteriophages (chronologically as discovered)—updated 12.2023.

N°	Bacteriophage Species	Virus Class, Morphotype	GenBank Accession Number	Host (Used for Propagation)	Genome			Original Discovery Reference	Isolate Location	Life Cycle	Growth Temperature (°C, Optimal/Range) and pH (Optimal/Range)
					Type and Size [bp]	ORFs	G+C [%]				
1	‘thermophilic lytic principle’	ND	ND	*B. stearothermophilus* T60	ND	ND	ND	Koser, 1926 [12]	sewage, polluted river water (USA)	lytic	52–60, app. 7
2	‘thermophilic bacteriophage’	ND	ND	ND	ND	ND	ND	Adant, 1928 [13]	ND	lytic	52–55
3	‘thermophilic bacteriophage’	ND	ND	*thermophilic bacterium* no. 10	ND	ND	ND	White et al., 1954, 1955 [14,15]	greenhouse soil (USA)	lytic	65 (50–70), 7
4	‘thermophilic bacteriophage’	ND	ND	*Bacillus* sp.	DNA, RNA (RNA is questionable)	ND	ND	Onodera 1961 [16]	compost (Japan)	lytic	65 (55–70), 7.2
5	TP-84	*Caudoviricetes*, *Saundersvirus*, *Siphovirus morphotype*	KY565347.1	*B. stearothermophilus* strain 10	circular, dsDNA, 47,718 bp	81	54.5	Saunders et al., 1964 [17] Saunders & Campbell, 1966 [18]	greenhouse soil (USA)	lytic	58 (43–76), 7.2
6	φ μ-4	ND	ND	*B. stearothermophilus*	ND	ND	ND	Shafia & Thompson, 1964 [19]	ND	lytic/lysogenic	50–65, 7
7	TP-1	*Siphovirus morphotype* (putative)	ND	*B. stearothermophilus*	dsDNA, 18,516 bp, (MW app. 12.1 MDa)	ND	42	Welker & Campbell, 1965 [20,21]	ND	lysogenic/lytic	55 (50–65), 7
8	ST1	*Siphovirus* *Myovirus* *morphotype* (putative)	ND	*B. stearothermophilus* strain S13	dsDNA	ND	43	Carnevali & Donelli, 1968 [22]	ND	lytic	60, app. 7
9	Tφ3	*Siphovirus morphotype*	ND	*B. stearothermophilus* ATCC 8005 S^R^	ds DNA, app. 35,700 bp (MW app. 23.2 MDa)	ND	40.2	Egbert & Mitchel, 1967 [23] Egbert, 1969 [24]	soil (USA)	lytic	60, 7.3
10	GH5	ND	ND	*B. stearothermophilus* NCA 1518	ND	ND	ND	Humbert & Fields, 1972 [25]	greenhouse soil (USA)	lytic	42.5–67, app. 7
11	GH8	*Siphovirus* *morphotype*	ND	*B. stearothermophilus* NCA 1518	ND	ND	ND	Humbert & Fields, 1972 [25]	greenhouse soli (USA)	lytic	42.5–67, app. 7
12	PhB1	*Siphovirus* *morphotype*	ND	*Bacillus* sp. strain B	ND	ND	ND	Ljunger & Edebo, 1972 [26]	farm soil (Sweden)	lytic	55, 7.3
13	D5	ND	ND	*B. stearothermophilus* NRS T91, ATCC 7953	ND	ND	ND	Reanney & Marsch, 1973 [27]	ND	lytic	45 (30–55), app. 7
14	D6	ND	ND	*B. stearothermophilus* NRS T91, ATCC 7953	ND	ND	ND	Reanney & Marsch, 1973 [27]	ND	lytic	45 (30–55), app. 7
15	D7	ND	ND	*B. stearothermophilus* NRS T91, ATCC 7953	ND	ND	ND	Reanney & Marsch, 1973 [27]	ND	lytic	45 (30–55), app. 7
16	D8	ND	ND	*B. stearothermophilus* NRS T91, ATCC 7953	ND	ND	ND	Reanney & Marsch, 1973 [27]	ND	lytic	45 (30–55), app. 7
17	φNS11	*Sphaerolipovirus* *morphotype* (putative)	ND	*B.acidocaldarius* TA6	dsDNA	ND	ND	Sakaki & Oshima, 1976 [28]	hot spring (Beppu, Japan)	lytic	60, 3.5 (2–5)
18	JS001	ND	ND	*B. caldotenax*	dsDNA	ND	ND	Sharp et al., 1986 [29]	ND	lytic/lysogenic	55 (50–70), 7.3 ± 0.2
19	JS004	ND	ND	*Bacillus thermophile* RS 239	dsDNA	ND	ND	Sharp et al., 1986 [29]	silage	lytic	55 (50–70), 7.3 ± 0.2
20	JS005	ND	ND	*B. thermophile* RS 239	dsDNA	ND	ND	Sharp et al., 1986 [29]	rotting straw	lytic	55 (50–70), 7.3 ± 0.2
21	JS006	ND	ND	*Bacillus thermophile* RS 239	dsDNA	ND	ND	Sharp et al., 1986 [29]	compost	lytic	55 (50–70), 7.3 ± 0.2
22	JS007	ND	ND	*Bacillus thermophile* RS 240	dsDNA	ND	ND	Sharp et al., 1986 [29]	silage	lytic	55 (50–70), 7.3 ± 0.2
23	JS008	ND	ND	*Bacillus thermophile* RS 241	dsDNA	ND	ND	Sharp et al., 1986 [29]	rotting straw	lytic	55 (50–70), 7.3 ± 0.2
24	JS009	ND	ND	*Bacillus thermophile* RS 242	dsDNA	ND	ND	Sharp et al., 1986 [29]	stable manure	lytic	55 (50–70), 7.3 ± 0.2
25	JS010	ND	ND	*Bacillus thermophile* RS 242	dsDNA	ND	ND	Sharp et al., 1986 [29]	compost	lytic	55 (50–70), 7.3 ± 0.2
26	JS011	ND	ND	*Bacillus thermophile* RS 239	dsDNA	ND	ND	Sharp et al., 1986 [29]	silage	lytic	55 (50–70), 7.3 ± 0.2
27	JS012	ND	ND	*Bacillus thermophile* RS 239	dsDNA	ND	ND	Sharp et al., 1986 [29]	compost	lytic	55 (50–70), 7.3 ± 0.2
28	JS013	ND	ND	*B. stearothermophilus* NCA 1503	dsDNA	ND	ND	Sharp et al., 1986 [29]	soil	lytic	55 (50–70), 7.3 ± 0.2
29	JS014	ND	ND	*B. stearothermophilus* NCA 1503	dsDNA	ND	ND	Sharp et al., 1986 [29]	rotting straw	lytic	55 (50–70), 7.3 ± 0.2
30	JS015	ND	ND	*B. stearothermophilus* NCA 1503	dsDNA	ND	ND	Sharp et al., 1986 [29]	compost	lytic	55 (50–70), 7.3 ± 0.2
31	JS017	ND	ND	*B. caldotenax*	dsDNA	ND	ND	Sharp et al., 1986 [29]	compost	lytic	55 (50–70), 7.3 ± 0.2
32	JS018	ND	ND	*B. caldotenax*	dsDNA	ND	ND	Sharp et al., 1986 [29]	rotting vegetation	lytic	55 (50–70), 7.3 ± 0.2
33	JS019	ND	ND	*B. caldotenax*	dsDNA	ND	ND	Sharp et al., 1986 [29]	rotting vegetation	lytic	55 (50–70), 7.3 ± 0.2
34	JS020	ND	ND	*B. caldotenax*	dsDNA	ND	ND	Sharp et al., 1986 [29]	rotting vegetation	lytic	55 (50–70), 7.3 ± 0.2
35	JS021	ND	ND	*B. caldotenax*	dsDNA	ND	ND	Sharp et al., 1986 [29]	rotting vegetation	lytic	55 (50–70), 7.3 ± 0.2
36	JS022	ND	ND	*B. caldotenax*	dsDNA	ND	ND	Sharp et al., 1986 [29]	compost	lytic	55 (50–70), 7.3 ± 0.2
37	JS023	ND	ND	*B. caldotenax*	dsDNA	ND	ND	Sharp et al., 1986 [29]	compost	lytic	55 (50–70), 7.3 ± 0.2
38	JS024	ND	ND	*B. caldotenax*	dsDNA	ND	ND	Sharp et al., 1986 [29]	compost	lytic	55 (50–70), 7.3 ± 0.2
39	JS025	ND	ND	*B. caldotenax*	dsDNA	ND	ND	Sharp et al., 1986 [29]	compost	lytic	55 (50–70), 7.3 ± 0.2
40	JS026	ND	ND	*B. caldotenax*	dsDNA	ND	ND	Sharp et al., 1986 [29]	compost	lytic	55 (50–70), 7.3 ± 0.2
41	JS027	ND	ND	*Bacillus thermophile* RS 241	dsDNA	ND	ND	Sharp et al., 1986 [29]	compost	lytic	55 (50–70), 7.3 ± 0.2
42	BVW1 (W1)	*Siphovirus morphotype*	ND	*Bacillus* sp. w13	dsDNA, app. 18 kb	ND	ND	Liu et al., 2006 [30]	deep-sea hydrothermal fields (West Pacific)	lytic	60, 7.0
43	GVE1 (E1)	*Siphovirus morphotype*	ND	*Geobacillus* sp. E 26323	dsDNA, app. 41 kb	ND	ND	Liu et al., 2006 [30]	deep-sea hydrothermal fields (East Pacific)	lytic	60, 7.0
44	GVE2 (E2)	*unclassified Caudoviricetes*, *Siphovirus* *morphotype*	NC_009552.3 DQ453159	*Geobacillus* sp. E 263	linear, dsDNA, 40,863 bp	62	44.8	Liu & Zhang, 2008 [31]	deep sea (China)	lysogenic	65, 7.0
45	GBSV1	*Caudoviricetes*, *Svunavirus*	NC_008376	*Geobacillus* sp. 6k512	linear, dsDNA, 34,683 bp	54	44.4	Liu et al., 2009, 2010 [32,33]	off-shore hot spring (Xiamen, China)	lytic	65, 7.2
46	BV1	*Caudoviricetes*, *Svunavirus sv1*	NC_009737, DQ840344	*Geobacillus* sp. 6k512	linear, dsDNA, 35,055 bp	54	44.4	Liu et al., 2009, 2010 [32,33]	off-shore hot spring (Xiamen, China)	lytic	65, 7.2
47	D6E	*unclassified Caudoviricetes*	NC_019544	*Geobacillus* sp. E 26323	circular, dsDNA, 49,335 bp	49	46	Wang & Zhang, 2010 [34]	deep-sea hydrothermal fields (East Pacific)	lytic	65, 7.0
48	AcaML1	*unclassified Caudoviricetes*, *Myovirus* *morphotype*	JX507079	*Acidithiobacillus caldus* ATCC 51756	dsDNA 59,363 bp	72	64.5	Tapia et al., 2012 [35]	coal spoil enrichment culture (Kingsbury, UK)	lytic/lysogenic	45 2.5
49	ϕOH2 (phiOH2)	*unclassified Caudoviricetes*, *Siphovirus morphotype*	AB823818, NC_021784	*G. kaustophilus* GBlys, *G. kaustophilus* NBRC 102445(T), lysogenic *G. kaustophilus* GBlys)	dsDNA, 38,099 bp	60	45	Doi et al., 2013 [36]	hot spring sediment (Japan)	lytic/lysogenic	55
50	GBK2	*unclassified Caudoviricetes*, *Siphovirus morphotype*	KJ159566	*G. kaustophilus*	circularly permuted, dsDNA, 39,078 bp	62	43	Marks & Hamilton, 2014 [37]	compost (Cary, NC, USA)	lytic	55, 7.3
51	GVE3 (E3)	*unclassified Caudoviricetes*, *Siphovirus morphotype*	NC_029073, KP144388	*G. thermoglucosidasius*	dsDNA 141,298 bp	202	29.6	Van Zyl et al., 2015 [38]	ND	lytic/lysogenic	60, 7.3
52	AP45	*Caudoviricetes*; *Kamchatkavirus*, *Siphovirus morphotype*	KX965989	*Aeribacillus* sp. CEMTC656	dsDNA 51,606 bp	71	38.3	Morozowa et al., 2019 [39]	soil (Valley of Geysers, Kamchatka, Russia)	lytic/lysogenic	55, 7.5
53	vB_Bps-36	*unclassified Caudoviricetes*	MH884513	*B. pseudalcaliphilus*	dsDNA 50,485 bp	68	41.1	Akhwale et al., 2019 [40]	Lake Elmenteita (Kenya)	lytic/?	30–40< 9<
54	vB_BpsM-61	*unclassified Caudoviricetes*	MH884514	*B. pseudofirmus* (*Alkalihalophilus pseudofirmus*)	dsDNA 48,160 bp	75	43.5	Akhwale et al., 2019 [40]	Lake Elmenteita (Kenya)	lytic/?	30–40< 9<
55	vB_BboS-125	*Caudoviricetes*; *Elmenteitavirus*	NC_048735.1 MH884509	*B. bogoriensis* (*Alkalihalobacillus borgiensis*)	dsDNA 58,528 bp	81	48.6	Akhwale et al., 2019 [40]	Lake Elmenteita (Kenya)	lytic/?	30–40< 9<
56	vB_BcoS-136	*unclassified Caudoviricetes*	MH884508	*B. cohnii* (*Sutcliffiella cohnii*)	dsDNA 160,590 bp	240	32.2	Akhwale et al., 2019 [40]	Lake Elmenteita (Kenya)	lytic/?	30–40< 9<
57	vB_BpsS-140	*unclassified Caudoviricetes*	MH884512	*B. pseudalcaliphilus* (*Alkalihalobacillus pseudalcaliphilus*)	dsDNA 55,091 bp	68	39.8	Akhwale et al., 2019 [40]	Lake Elmenteita (Kenya)	lytic/?	30–40< 9<
58	GR1	*unclassified Caudoviricetes*, *Siphovirus morphotype*	OK896991	*G. stearothermophilus* ATTC 10149	dsDNA 79,387 bp	108	32.34	Choi &Kong, 2023 [41]	soil Gyeongchun Line railroad (Seul, Republic of Korea)	lytic	50 7.2
59	vB_GthS_PT9.1	*unclassified Caudoviricetes*, *Siphovirus morphotype*	OP341630	*G. thermodenitrificans*	dsDNA 38,373 bp	75	43.9	Šimoliūnas et al., 2023a [42]	compost heaps (Lithuania)	lytic	45–80
60	vB_GthS_NIIg9.7	*unclassified Caudoviricetes*, *Siphovirus morphotype*	OP341624	*G. thermodenitrificans* NIIg-9	dsDNA 39,016 bp	76	44.4	Šimoliūnas et al., 2023a [42]	compost heaps (Lithuania)	lytic	50–78
61	vB_GthS_PK5.1	*unclassified Caudoviricetes*, *Siphovirus morphotype*	OP341628	*G. thermodenitrificans*	dsDNA 38,161 bp	64	43.6	Šimoliūnas et al., 2023a [42]	compost heaps (Lithuania)	lytic	48–80
62	vB_GthS_PK3.5	*unclassified Caudoviricetes*, *Siphovirus morphotype*	OP341626	*G. thermodenitrificans*	dsDNA 38,788 bp	76	43.5	Šimoliūnas et al., 2023a [42]	compost heaps (Lithuania)	lytic	50–78
63	vB_GthS_PK3.6	*unclassified Caudoviricetes*, *Siphovirus morphotype*	OP341627	*G. thermodenitrificans*	dsDNA 38,405 bp	68	44.8	Šimoliūnas et al., 2023a [42]	compost heaps (Lithuania)	lytic	50–80
64	vB_PtoS_NIIg3.2	*unclassified Caudoviricetes*, *Siphovirus morphotype*	OP341623	*P. toebii strain NIIg-3* *G. thermodenitrificans*	dsDNA 38,970 bp	42.2	42.2	Šimoliūnas et al., 2023b [43]	compost heaps (Lithuania)	lytic/lysogenic?	50–80
65	vB_GthS_PK5.2,	*unclassified Caudoviricetes*, *Siphovirus morphotype*	OP341629.1	*G. thermodenitrificans*	dsDNA	ND	ND	Šimoliūnienė et al., unpublished data [44]	compost heaps (Lithuania)	lytic	ND
66	vB_GthS_PK2.1,	*unclassified Caudoviricetes*, *Siphovirus morphotype*	OP341625.1	*G. thermodenitrificans*	dsDNA	ND	ND	Šimoliūnienė et al., unpublished data [44]	compost heaps (Lithuania)	lytic	ND
67	vB_GthS_NIIg10.1,	*unclassified Caudoviricetes*, *Siphovirus morphotype*	ND	*G. thermodenitrificans*	dsDNA	ND	ND	Šimoliūnienė et al., unpublished data [44]	compost heaps (Lithuania)	lytic	ND
68	vB_GthS_NIIg2.1,	*unclassified Caudoviricetes*, *Siphovirus morphotype*	ND	*G. thermodenitrificans*	dsDNA	ND	ND	Šimoliūnienė et al., unpublished data [44]	compost heaps (Lithuania)	lytic	ND
69	vB_GthS_NIIg2.2,	*unclassified Caudoviricetes*, *Siphovirus morphotype*	ND	*G. thermodenitrificans*	dsDNA	ND	ND	Šimoliūnienė et al., unpublished data [44]	compost heaps (Lithuania)	lytic	ND
70	vB_GthS_NIIg2.3	*unclassified Caudoviricetes*, *Siphovirus morphotype*	ND	*G. thermodenitrificans*	dsDNA	ND	ND	Šimoliūnienė et al., unpublished data [44]	compost heaps (Lithuania)	lytic	ND
71	A403	*Caudoviricetes*; *Tandoganvirus*	NC_048701 MG969427	*Anoxybacillus caldiproteolyticus*	dsDNA 40,847 bp	ND	ND	Sahin et al., unpublished data [44]	ND	ND	ND
72	JGon-2020a	ND	CP063417	*P.* *thermoglucosidasius* 23.6	dsDNA 55,505 bp	ND	ND	Delgado & Gonzalez, unpublished data [45]	ND	ND	ND

ND—not determined; ORFs—Open Reading Frames; dsDNA—double-stranded DNA.

**Table 2:** New (this work) experimentally confirmed functional CDSs of bacteriophage TP-84 genome (updated December 2023).

CDS Name	CDS Length (bp)	Location in the Genome (bp)	CDS Arbitrary Orientation	Polypeptide Length (aa)	Predicted Polypeptide Molecular Weight (kDa)	Experimentally Determined Polypeptide Molecular Weight (kDa)	Predicted Isoelectric Point	Hypothetical Function (Analysis)	Confirmed by Proteomic Analysis
TP84_03	687	1868–2554	+	228	26.3	26.3	9.17	DUF3310 domain-containing protein	unknown
TP84_07	147	4799–4945	+	48	5.3	ND	7.94	unknown	unknown
TP84_09	210	7004–7213	+	69	8.2	ND	4.89	putative membrane-associated protein	unknown
TP84_10	828	7441–8268	+	275	31.6	ND	4.70	putative prohead protease	putative prohead protease
TP84_14	330	10,032–10,361	+	109	12.2	ND	5.16	unknown	unknown
TP84_17	378	11,162–11,539	+	125	14.6	14.6	5.35	tail assembly protein	tail assembly protein
TP84_24	210	19,061–19,270	+	69	7.9	ND	4.59	unknown	unknown
TP84_28	1185	23,676–24,860	+	394	44.2	44.2	9.67	endolysin	endolysin
TP84_31	156	26,023–26,178	+	51	6.0	ND	6.15	putative membrane protein	putative membrane protein
TP84_32	255	26,235–26,489	+	84	9.8	9.7	9.70	unknown	unknown
TP84_34	255	26,884–27,138	+	84	9.6	ND	4.48	putative membrane protein	putative membrane protein
TP84_36	237	27,353–27,589	+	78	9.3	9.3	9.91	unknown	unknown
TP84_37	396	27,698–28,093	+	131	14.9	14.8	6.41	unknown	unknown
TP84_38	201	28,090–28,290	+	66	7.4	7.4	6.73	unknown	unknown
TP84_39	216	28,403–28,618	+	71	8,1	ND	7.96	unknown	unknown
TP84_40	210	28,615–28,824	+	69	8.2	ND	9.39	unknown	unknown
TP84_44	132	29,708–29,839	+	43	5.0	ND	10.62	unknown	unknown
TP84_45	231	29,839–30,069	+	76	8.5	ND	9.75	unknown	unknown
TP84_50	159	33,468–33,626	+	52	5.8	ND	6.53	unknown	unknown
TP84_51	159	33,832–33,990	+	52	6.1	ND	10.41	aspartyl-phosphate phosphatase Spo0E family protein	unknown
TP84_52	237	34,030–34,266	+	78	8.5	ND	5.36	transcriptional regulator (HTH_XRE family)	transcriptional regulator (HTH_XRE family)
TP84_53	1017	34,238–35,254	+	338	38.5	ND	5.33	RecB-like protein	RecB-like protein
TP84_54	1020	35,270–36,289	+	339	38.8	ND	5.78	DNA single-strand annealing protein	DNA single-strand annealing protein
TP84_55	222	36,337–36,558	+	73	8.8	ND	4.54	unknown	unknown
TP84_56	183	36,924–37,106	+	60	7.4	7.4	9.25	unknown	unknown
TP84_57	783	37,127–37,909	+	260	30.3	30.4	8.57	conserved phage C-terminal domain-containing protein (bacterial)	unknown
TP84_63	468	40,851–41,318	+	155	17.4 (18.3) *	17.4 (18.3) *	5.16	single-stranded DNA-binding protein	single-stranded DNA-binding protein
TP84_64	240	41,343–41,582	+	79	9.3	9.3	6.89	unknown	unknown
TP84_66	270	41,751–42,020	+	89	10.4	ND	5.92	single-stranded DNA-binding protein	single-stranded DNA-binding protein
TP84_67	183	42,023–42,205	+	60	6.9	ND	4.58	unknown	unknown
TP84_68	987	42,209–43,195	+	328	38.6	38.5	6.91	thymidylate synthase	thymidylate synthase
TP84_69	501	43,200–43,700	+	166	20.0	20.1	5.67	dUTP diphosphatase	dUTP diphosphatase
TP84_71	246	44,014–44,259	+	81	9.4	ND	10.21	unknown	unknown
TP84_72	504	44,246–44,749	+	167	19.3	ND	7.70	Holliday junction-specific endonuclease	Holliday junction-specific endonuclease
TP84_76	126	45,675–45,800	+	41	4.8	ND	5.54	unknown	unknown
TP84_77	186	45,857–46,042	+	61	7.2	ND	9.19	unknown	unknown
TP84_78	204	46,047–46,250	+	67	7.5	ND	12.16	unknown	unknown
TP84_79	354	46,380–46,733	+	117	13.4	13.4	7.82	unknown	unknown
TP84_80	366	46,741–47,106	+	121	14.0	13.9	6.82	unknown	unknown

ND—not determined; CDs—coding sequences; * SDS-PAGE molecular weight determination of the recombinant version of SSB-His-tag protein.

## Supplementary Materials

The following supporting information can be downloaded at: https://www.mdpi.com/article/10.3390/ijms25063125/s1.
