# Supplementary material for: Bacteriophages of Thermophilic ‘Bacillus Group’ Bacteria—A Systematic Review, 2023 Update
Source: Int J Mol Sci. 2024 Mar 8;25(6):3125. doi: 10.3390/ijms25063125 (PMC10969951; doi:10.3390/ijms25063125)
Supplement: Supplementary file 1 [file ijms-25-03125-s001.zip › ijms-2866891-supplementary.pdf]

Supplementary files

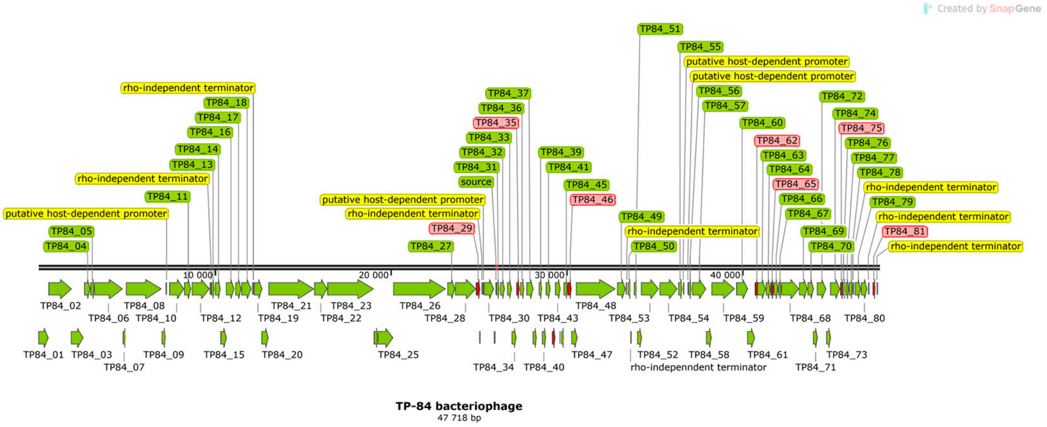

Figure S1. Genome map of TP-84 bacteriophage, showing updated proteomic confirmation of the bioinformatically characterized ORFs [51, this work]. The color code is as follows: green – ORFs confirmed as active in proteins biosynthesis; red – ORFs unconfirmed to date (12.2023) as active in proteins biosynthesis.

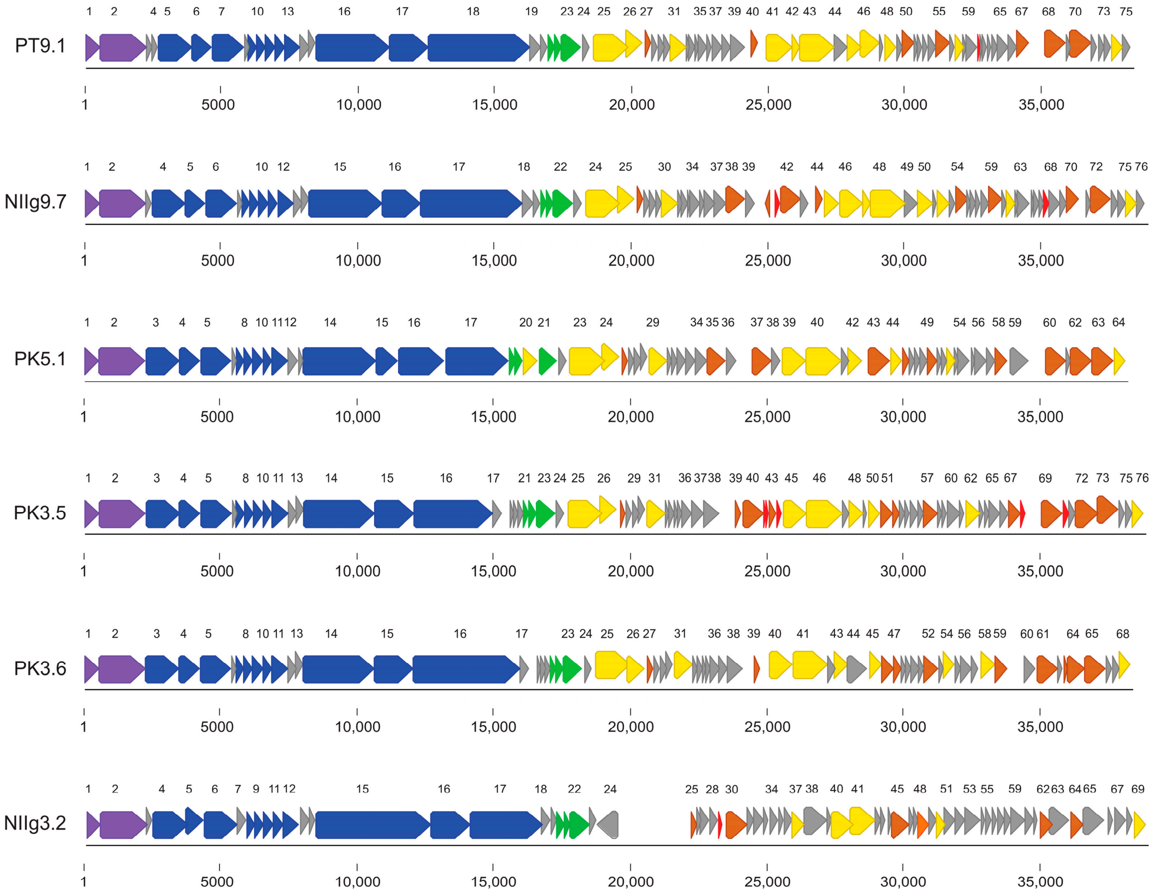

Figure S2. Functional genome maps of *(Para)geobacillus* bacteriophages. The coding capacity of the genomes are shown. Numbers indicate ORF position in genome, functions are assigned according to the characterized ORFs in NCBI database and HHpred analysis. The color code is as follows: yellow—DNA replication, recombination, and repair; blue—structural proteins, phage–host interactions; purple—DNA packaging; brown—transcription, translation, nucleotide metabolism; light green—lysis; dark green—lysogeny; orange—auxiliary metabolic genes; grey—conserved hypothetical proteins; red— hypothetical proteins with no reliable identity when compared to database entries.
